# Supplementary material for: Clinical Practice Use of Liquid Biopsy to Identify RAS/BRAF Mutations in Patients with Metastatic Colorectal Cancer (mCRC): A Single Institution Experience
Source: Cancers (Basel). 2019 Oct 8;11(10):1504. doi: 10.3390/cancers11101504 (PMC6827157; doi:10.3390/cancers11101504)
Supplement: Supplementary file 1 [file cancers-11-01504-s001.pdf]

Clinical Practice Use of Liquid Biopsy to Identify *RAS/BRAF* Mutations in Patients with Metastatic Colorectal Cancer (mCRC): A Single Institution Experience

Pietro Paolo Vitiello, Vincenzo De Falco, Emilio Francesco Giunta, Davide Ciardiello, Claudia Cardone, Pasquale Vitale, Nicoletta Zanaletti, Carola Borrelli, Luca Poliero, Marinella Terminiello, Gianluca Arrichiello, Vincenza Caputo, Vincenzo Famiglietti, Valentina Mattera Iacono, Francesca Marrone, Alessandra Di Liello, Giulia Martini, Stefania Napolitano, Michele Caraglia, Angela Lombardi, Renato Franco, Ferdinando De Vita, Floriana Morgillo, Teresa Troiani, Fortunato Ciardiello and Erika Martinelli

Table S1. Patients' clinical characteristics across the three cohorts used in the analyses.

Patients' Characteristics	Global Cohort *n* (%)	Basal Cohort *n* (%)	Post-EGFR Cohort *n* (%)
**Sex:**			
Male	36(50%)	20(47%)	16(53%)
Female	36(50%)	22(53%)	14(47%)
**Age:**			
<65	34(47%)	19(45%)	15(50%)
≥65	38(53%)	23(55%)	15(50%)
**ECOG Performance status:**			
0	46(64%)	27(64%)	19(63%)
1	18(25%)	11(26%)	7(24%)
≥2	8(11%)	4(10%)	4(13%)
**Primary tumour:**			
Right colon (including transverse)	16(22%)	11(26%)	5(17%)
Left colon	40(56%)	18(43%)	22(73%)
Rectum	16(22%)	13(31%)	3(10%)
**Metastatic sites:**			
Liver	49(68%)	26(62%)	23(77%)
Lungs	32(44%)	18(43%)	14(47%)
Nodes	40(56%)	21(50%)	19(63%)
Peritoneum	14(19%)	11(26%)	5(17%)
Others	12(17%)	5(12%)	7(23%)
**N° of metastatic sites:**			
<3	52(72%)	34(81%)	18(60%)
≥3	20(28%)	8(19%)	12(40%)
**LDH (lactate dehydrogenase):**			
<ULN	38(53%)	26(62%)	12(40%)
≥ULN	34(47%)	16(38%)	18(60%)
**ALP (alkaline phosphatase):**			
<ULN	41(57%)	27(64%)	16(53%)
≥ULN	31(43%)	17(36%)	14(47%)

© 2019 by the authors. Licensee MDPI, Basel, Switzerland. This article is an open access article distributed under the terms and conditions of the Creative Commons Attribution (CC BY) license (http://creativecommons.org/licenses/by/4.0/).
